# Speech and Non-Speech Audio-Visual Illusions: A Developmental Study

**DOI:** 10.1371/journal.pone.0000742

**Published:** 2007-08-15

**Authors:** Corinne Tremblay, François Champoux, Patrice Voss, Benoit A. Bacon, Franco Lepore, Hugo Théoret

**Affiliations:** 1 Department of Psychology, University of Montreal, Montreal, Canada; 2 Research Center, Sainte-Justine Hospital, Montreal, Canada; 3 Speech Language Pathology and Audiology, University of Montreal, Montreal, Canada; 4 Department of Psychology, Bishop's University, Sherbrooke, Quebec, Canada; University of Sydney, Australia

## Abstract

It is well known that simultaneous presentation of incongruent audio and visual stimuli can lead to illusory percepts. Recent data suggest that distinct processes underlie non-specific intersensory speech as opposed to non-speech perception. However, the development of both speech and non-speech intersensory perception across childhood and adolescence remains poorly defined. Thirty-eight observers aged 5 to 19 were tested on the McGurk effect (an audio-visual illusion involving speech), the Illusory Flash effect and the Fusion effect (two audio-visual illusions not involving speech) to investigate the development of audio-visual interactions and contrast speech vs. non-speech developmental patterns. Whereas the strength of audio-visual speech illusions varied as a direct function of maturational level, performance on non-speech illusory tasks appeared to be homogeneous across all ages. These data support the existence of independent maturational processes underlying speech and non-speech audio-visual illusory effects.

## Introduction

It has repeatedly been shown that intersensory redundancy, the congruent bimodal presentation of stimuli over two sensory modalities, can enhance perception in both modalities (e.g. [1], [2]). It is also well established that when two sensory modalities convey incongruent information (i.e. non-specific intersensory effects; [3]), accuracy of perception can suffer. In the McGurk effect [4], vision biases audition. In this classic demonstration based on the perception of spoken syllables, incongrent lip movements induce the misperception of auditory inputs. For example, upon hearing/baba/but seeing/gaga/, most subjects will report hearing the fused percept/dada/[4]. Subsequent studies have confirmed that the McGurk effect is a very robust illusion [5], [6]. Although vision was first thought to dominate audio-visual interactions [7], more recent findings suggest that auditory inputs can also bias visual perception. In the “Illusory Flash effect” or “sound-induced flashing” [8] a single visual flash can be perceived as two flashes if it is accompanied by two (rather than one) successive sounds. Conversely, in the “Fusion effect” [9] two physical flashes can be fused as one if they are accompanied by a single auditory signal.

Based on these findings, theoretical accounts relating how the senses interact to create a unified percept have emerged [3], [10]. It has recently been suggested that different mechanisms could underlie speech as opposed to non-speech interaction effects. Indeed, in adult observers, audio-visual interaction is stronger when a set of identical stimuli is treated as speech rather than non-speech; this supports a “speech-specific mode of perception” [11]. At the physiological level, intersensory speech and non-speech interactions also appear to rely, at least in part, on distinct mechanisms. McGurk-type illusory effects recruit the posterior parietal cortex around 150 ms before activating occipital areas at around 270 ms [12]. In the Illusory Flash effect, modulation of the visual cortex occurs much earlier (∼150 ms; [13]). Functional imaging data also show that intersensory interactions rely on multiple brain areas that are differentially involved in the intersensory process (for a review, see [14]). For example, parts of the superior temporal sulcus have been repeatedly shown to play an important role in object recognition, including recognition of audio-visual speech information, whereas audio-visual spatial processing has predominantly been associated with activation of the intraparietal sulcus [15]–[17].

Although speech and non-speech intersensory effects have been well characterized in adult observers, developmental patterns remain poorly understood. McGurk-type illusory phenomena have been studied in infants [18]–[20] and children [4], [21], [22] but no study has used an age range sufficiently broad to map the developmental course of this phenomenon. Moreover, to our knowledge no study has attempted to map the developmental course of non-specific, non-speech intersensory effects in childhood and adolescence. Indeed, the few studies that touched on intersensory perception in children have centered on their ability to perceive intersensory *equivalence* (see [3]). Finally, to our knowledge, no study has yet simultaneously assessed both speech and non-speech intersensory illusions in children and adolescents.

In the present study, speech (McGurk effect) and non-speech (Illusory Flash effect and Fusion) illusions were presented to the same observers across three age categories (5–9, 10–14 and 15–19 years old). Hence, we aimed at *i*) determining the presence of non-specific, non-speech intersensory effects at different developmental stages; and *ii*) describing and contrasting the developmental course of non-specific speech/non-speech illusory effects.

## Methods

Thirty-eight French-speaking subjects (15 males, 23 females) aged 5 to 19 years participated in the study. Each age (e.g. 9 years old) was represented by at least two participants. Three age groups were defined *a priori*: 5–9 (11 subjects), 10–14 (16 subjects), and 15–19 (11 subjects) years of age. The study was approved by the institutional Research Ethics Board of Hôpital Sainte-Justine and written informed consent was obtained from all participants and their parents. Individuals with a diagnosed or suspected neurodevelopmental disorder, attention deficit and hyperactivity disorder or learning disorder were excluded from the study. All participant had normal or corrected-to-normal vision as well as normal hearing.

Participants were seated in a semi-dark room with the head on a chin rest located 57 cm from the computer screen (and speakers) where the stimuli were presented. The McGurk effect, the Illusory Flash effect and the Fusion effect tasks were performed in a single session, in counterbalanced order. In all tasks, visual stimuli were presented either at fixation or 5 degrees below fixation. This procedure was implemented because the strength of at least one of the illusions used in the present study has been shown to be greater for parafoveal presentations (the Illusory Flash effect; [23]). Stimuli were presented on a 17-inch Viewsonic computer screen using a Powermac G4 computer (Apple Inc., Cuppertino, CA, USA). Stimuli were delivered with Psyscope for the McGurk effect and Matlab (The Mathworks Inc., Natick, MA, USA) for the Illusory Flash effect and Fusion effect. To ensure fixation and reject the trials in which fixation did not occur, eye movements were monitored on-line (EyeLink, SR Research, Mississauga, Canada).

### The McGurk effect

In the McGurk effect task, the voice of an adult male articulating syllables was presented in either a unimodal (auditory only) or bimodal manner. In bimodal trials, the auditory stimulus and the video of the articulatring face (subtending 5 degrees of visual angle) were presented simultaneously. In congruent trials, the auditory (voice) and visual (face) signals carried the same information whereas in incongruent trials, they did not. Five different experimental conditions were used: 1) unimodal auditory/va/; 2) unimodal auditory/ba/; 3) bimodal congruent/va/; 4) bimodal congruent/ba/; and 5) bimodal incongruent auditory/ba/and visual/va/. The bimodal and unimodal trials were repeated ten times each in random order.

Participants were instructed to look at a fixation cross that was presented at the center of the screen for 1000 ms before each trial. Immediately following the disappearance of the cross, a stimulus was presented. Observers were told to simply repeat the syllable they had heard as clearly and precisely as possible. A break was systematically offered at 3 different times during the experiment, but participants could also take a break at any moment if needed. All incorrect responses in the incongruent bimodal condition (anything other than/ba/) were considered manifestations of the McGurk effect.

After the McGurk effect task, a mute control task was performed in order to assess the participants' lip-reading abilities. In this task, the stimuli were unimodal visual/ba/and/va/lip movements. Again, the stimuli were presented at fixation and 5 degrees below fixation. Each condition was repeated 10 times for a total of 40 trials (2 stimuli × 2 locations × 10 trials).

### Illusory Flash effect and Fusion effect

The characteristics of the stimuli used in the Illusory Flash effect task and Fusion effect were similar to those used in Shams et al. [8], [13]. The flash was a white circle subtending 2 degrees of visual angle. It had a luminance of 0.02 cd/m. The auditory signal was made of one or two 7 ms beeps with a frequency of 3500 Hz.

Pilot trials revealed that the inter-flash delay of 67 ms used by Shams et al. [8] was too short for many children to be able to visually distinguish one from two flashes. A pre-experimental task was therefore conducted to determine the optimal inter-flash delay for each participant. The fastest delay between flashes in which the participant reached an efficiency score of at least 93% (15/16) was used in the experimental task. Eight conditions (number of flashes (2) X number of beeps (2) X location (2)) were presented in randomized order. Ten trials per condition were presented. Subjects were simply asked to judge the number of flashes that appeared on the screen (one or two).

## Results

### McGurk effect

**Figure 1:**
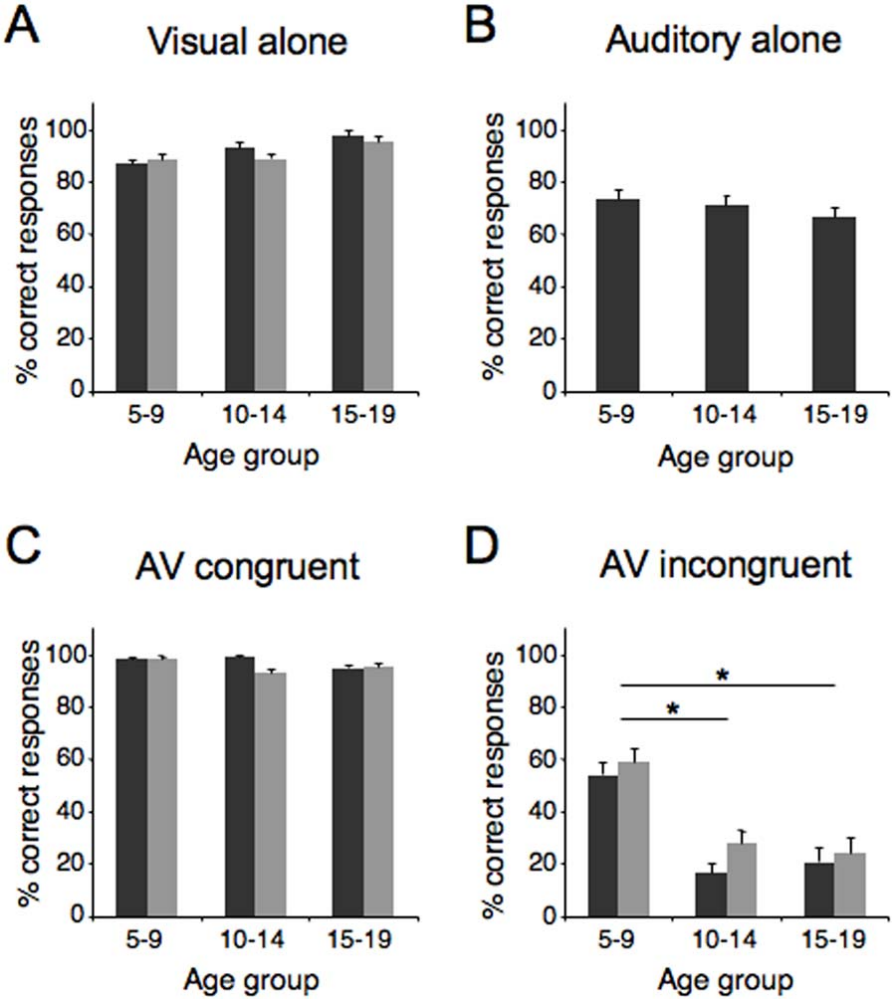
Subjects' performance on the McGurk effect. For visual trials (A), auditory trials (B) and congruent audiovisual trials (C), performance was similar across age groups. Performance in the incongruent trials (D) revealed that the 5–9 year-old group perceived significantly fewer McGurk illusions than the two older groups of children. Dark bars: peripheral visual presentation; Light bars: central visual presentation. Error bars represent between-subject SEM. * : p<0.05.

For visual-only trials (lip-reading), a 3×2 repeated measures ANOVA with *age* (5–9, 10–14, 15–19) as a between-subjects factor and *position* (center, periphery) as a within-subjects factor indicated that performance in control trials was homogeneous across age groups (F = 1.9, p = 0.15; Figure 1a). For auditory trials and congruent audiovisual trials, a one-way ANOVA with *age* as a between-subjects factor was conducted. Performance was similar across age groups for both auditory (F = 0.60, p = 0.45; Figure 1b) and congruent audiovisual (F = 1.17, p = 0.32; Figure 1c) conditions.

To determine the robustness of the McGurk effect across age groups, a 3×2 repeated measures ANOVA with *age* as a between-subjects factor and *position* as a within-subjects factor was performed on bimodal incongruent trials. There were main effects of *age* (F = 5.10, p = 0.01) and *position* (F = 4.11, p = 0.05) . The interaction between factors was not significant (F = 0.67, p = 0.52). Post hoc t-tests revealed that the 5–9 year-old group perceived significantly fewer McGurk illusions than the 10–14 (p = 0.02) and the 15–19 year-old groups (p = 0.04) (Figure 1d). In addition, more McGurk illusions were perceived when the visual stimuli were presented at fixation (p = 0.03).

**Figure 2:**
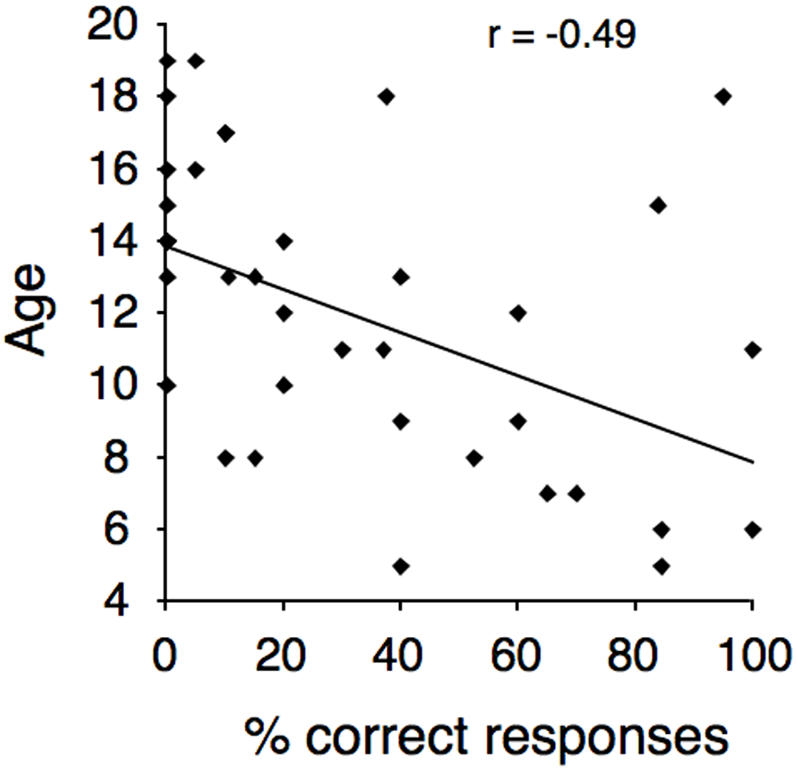
Percent of correct (non-biased) responses in the incongruent condition McGurk effect plotted as a function of age.

**Figure 3:**
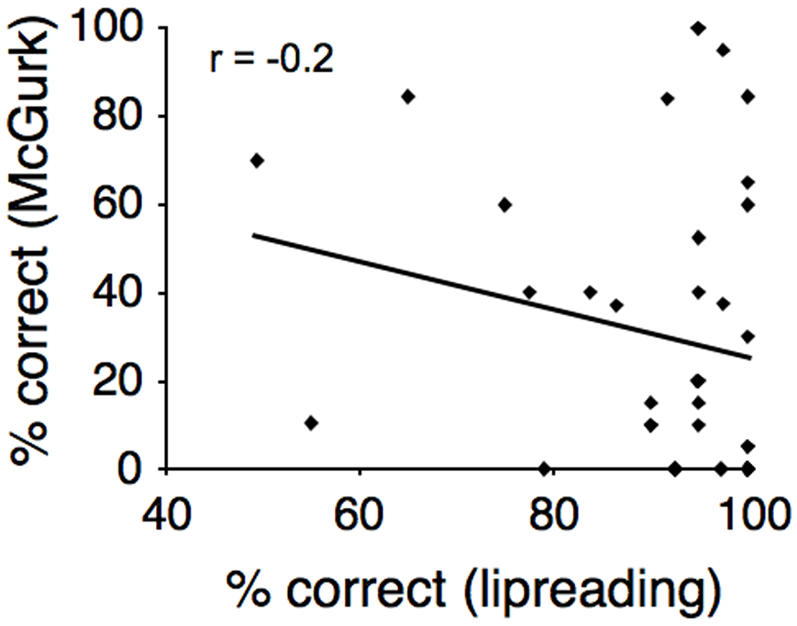
Percent of correct (non-biased) responses in the incongruent condition McGurk effect plotted as a function of lipreading ability.

To further test the effect of age on the McGurk effect, individual subjects' ages were correlated with the number of trials in which a McGurk illusion was perceived. A two-tailed Pearson correlation revealed significant effects in both central (r = −0.475, p = 0.003) and peripheral (r = −0.459, p = 0.004) locations, as well as when both these conditions were collapsed (r = −0.49, p = 0.002; Figure 2). Finally, to determine the influence of lip-reading ability on the integration of audio-visual speech cues, a correlation between participants' correct responses in the mute control task and the number of McGurk illusions was computed. The correlation was not significant (r = −0.2, p = 0.23; Figure 3).

### Illusory Flash effect and Fusion effect

**Figure 4:**
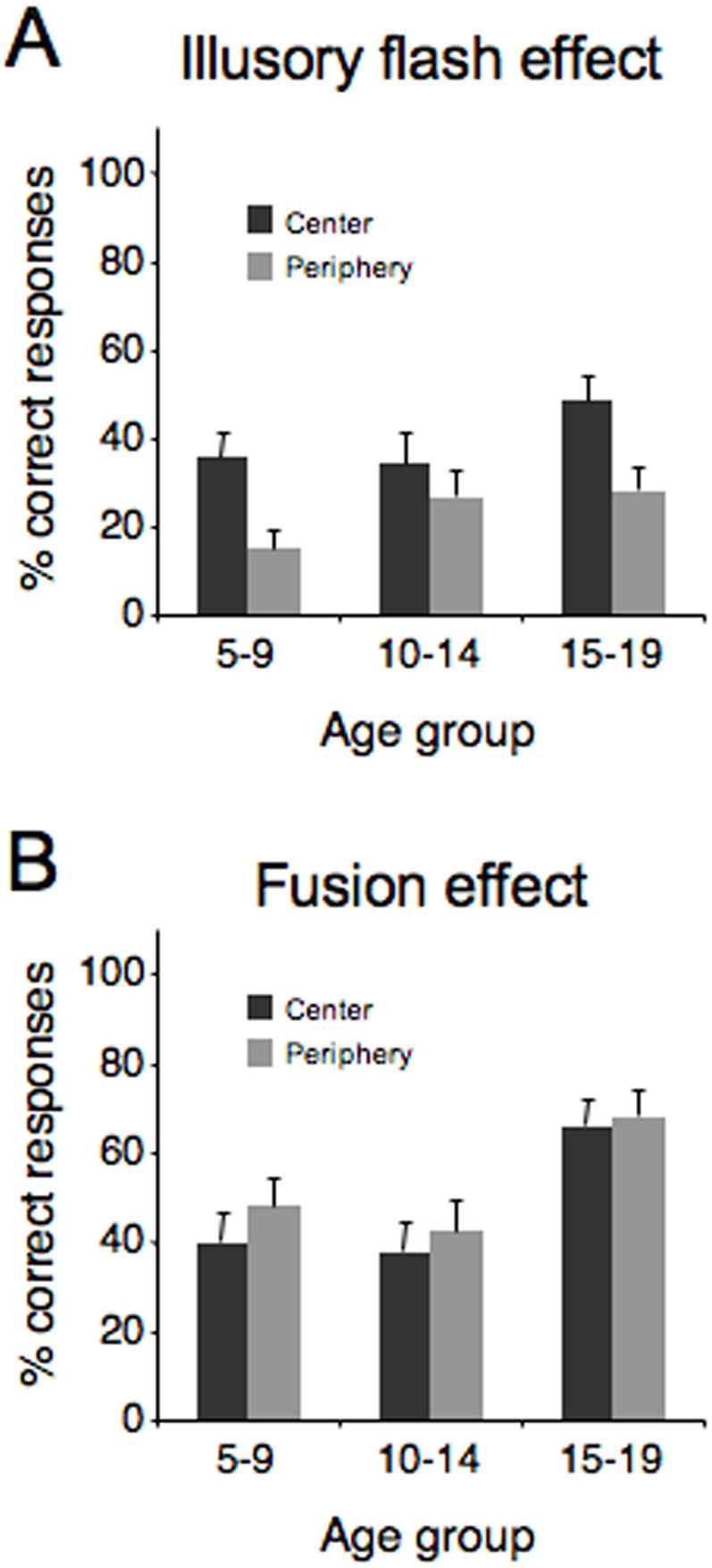
Subjects' performance on the Illusory Flash (A) and the Fusion (B) effects. For both illusory percepts, there was no effect of age. Error bars represent between-subject SEM.

The original illusion (Shams, 2000) was replicated as the number of correct responses in the 1 flash/2 beeps condition was drastically reduced (Figure 4a). A 3×2 repeated measures ANOVA with *age* (5–9, 10–14, 15–19) as a between-subjects factor and *position* (center, periphery) as a within-subjects factor revealed a main effect for *position* (F = 10.64, p = 0.002), but no main effect for *age* (F = 0.52, p = 0.60). The interaction was also non-significant (F = 0.74, p = 0.49). This is in line with previous work, where the Illusory Flash effect has been shown to be more robust at a perifoveal location (Shams et al., 2002). The strength of the illusion was not correlated with participant age (center: r = 0.12, p = 0.456; periphery: r = 0.25, p = 0.12). As for the Fusion effect (Figure 4b), there were no significant effects for either *age* (F = 1.81, p = 0.18) or *position* (F = 1.76, p = 0.19) and the interaction was non-significant (F = 0.22, p = 0.80).

There was no correlation between the Illusory Flash effect and the McGurk effect (center: r = −0.167, p = 0.32; periphery: r = −0.22, p = 0.182) or the Fusion effect and the McGurk effect (center: r = −0.28; periphery: r = −0.206, p = −0.21).

## Discussion

The purpose of this study was to investigate the developmental course of non-specific audio-visual effects on a maturational continuum. Our main finding is a discrepancy in the maturational patterns of speech and non-speech audio-visual effects.

Illusory percepts of audio-visual speech elements have been shown to occur in infants [18]–[20] but these are weaker and more inconsistent than what is observed in adults, suggesting that experience with speech may be an important component of audio-visual speech perception [20]. In pre-school and school-aged children, previous findings indicate that incongruent visual input has less influence on the final percept resulting from a McGurk illusion [4], [21], [22] and that when a single modality is chosen for the final bimodal percept in a McGurk illusion, children choose the auditory modality whereas adults choose vision [4], [21], [22]. Our results are consistent with and extend previous findings by showing that an important proportion of the maturational processes underlying speech intersensory effects is not completely developed before 10 years of age, since 5–9 year-olds presented a different pattern of intersensory speech effect in comparison with the two older groups. Indeed, the significant correlation between age and the frequency of illusory percepts suggests that audio-visual speech perception continues to evolve during childhood. Massaro et al. [22] have suggested that the weaker McGurk effect observed in young children is due to poorer lip-reading abilities. We found no significant difference in lip-reading abilities across the three age-groups. Although a ceiling effect in the older group of children may have prevented small lip-reading differences from being revealed, the absence of a significant correlation between lip-reading ability and the frequency of McGurk illusions argues against this explanation. In addition, Massaro and collaborators have suggested that lip-reading performance becomes similar to adults “sometime after the child's 6^th^ year” [22], a notion that is supported by a study showing that speech reading abilities become stable near 7 years of age [24]. Our data are in line with this interpretation and suggest that the weaker influence of visual input on bimodal speech perception in children that are more than 6 years old may be explained by the degree to which visual and speech cues are integrated.

To our knowledge, a single study has shown that non-speech illusions can occur in infants. In the “Streaming-Bouncing” effect [25], two disks move towards the centre of a screen. When the two disks cross in silence, they are perceived as passing through one another. However, when a sound is emitted as the disks meet they appear to bounce off each other. Using this effect, Scheier et al. [26] have shown that this non-specific intersensory capability emerges halfway through the first year of life. Thus, prior to the present investigation, non-speech audio-visual illusions have only been observed in a spatiotemporal task where audition biases vision. The developmental course of non-speech illusory percept remains uncharted. Our findings reveal a homogeneous profile for all ages for the two non-verbal tasks. Therefore, all age groups performed equally on both the Illusory Flash effect and the Fusion effect. These findings are consistent with the suggestion that audio-visual non-speech integration appears very early in life [26].

It is important to note that both illusion categories not only differ with respect to the speech/non speech content but also in the way participants respond. In the McGurk effect, children must report what they hear whereas in the two non-speech illusions they report what they see. Some have suggested that the strength of a single modality on perceptual judgment depends on the attention it is given [27], which in the present case could explain the different pattern of age-related differences in the two illusory categories. In a study of bimodal speech perception in 6 year old children, however, Massaro [21] showed that directing attention to the speaker's mouth did not modify the proportion of incorrect responses in a McGurk-like task. Electrophysiological data also support the idea that audiovisual integration is a preattentive phenomenon since a mismatch negativity can be evoked by McGurk-like stimuli [28]. As such, some authors have suggested that audiovisual speech perception is an automatic process (see [29] for a review). Conversely, it has been shown that responses to McGurk stimuli differ when participants are asked to respond to the visual or auditory cue [30] and directing attention away from the mouth area significantly reduces the strength of the McGurk effect [29]. Interestingly, contrary to audiovisual stimuli, unisensory responses in the McGurk task do not appear to be influenced by attentional shifts, suggesting that it is integration *per se* that varies with attention [29]. However, when data are fitted in a model of perception (Fuzzy Logical Model of Perception; [31]), predictions are that it is not the integration level that is affected by attention but unisensory processing [29]. These discrepancies highlight the fact it is still premature to ascertain whether it is only the speech/non speech distinction that separates performance on both types of illusions tested here. In addition to attention and modality of response, it may be that the different pattern of results reflects the fact that in young children vision may have less impact on hearing than in older children, whereas hearing has comparable effects on vision across all ages. In this case, the fact that vision biases audition in the McGurk effect and that audition biases vision in the illusory flash effect may also explain parts of the data. Nevertheless, our results clearly show that the McGurk illusion, which involves speech material, does not follow the same developmental rules than the illusory flash and fusion effects. Further studies are needed to specifically address which factors contribute to this difference, and to what extent.

Finally, the suggestion that speech and non-speech integration follow different developmental time courses does not exclude the possibility that they share common mechanisms. Indeed, it may be hypothesized that both illusory phenomena are subtended similarly at low hierarchical levels whereas audio-visual integration of speech elements requires supplementary processing. For example, it has been shown that brainstem structures are involved in *both* audio-visual speech [32], [33] and non-speech integration [34], [35], suggesting the existence of common substrates.
